# Pathological Anatomy

**Published:** 1860-11

**Authors:** John H. Packard

**Affiliations:** Philadelphia


### PATHOLOGICAL ANATOMY.

BY JOHN H. PACKARD, M.D., OF PHILADELPHIA.

I.—*Embolie of the Middle Cerebral Artery.* By Prof. LEBERT, of the University of Breslau.

A carpenter, aged twenty-five, had for some months had palpitations of the heart, and painful dyspnœa on going up stairs. He had never had acute articular rheumatism. Six weeks previous to his application at the medical clinique of the university, he had passed bloody urine, and had had pain about the kidneys.

The day before his admission, having cut his finger and lost a good deal of blood, he suddenly fell down insensible; he did not regain his consciousness for two* hours, and was then speechless, as well as paralyzed on the right side. Sensation was unimpaired. At the time of his admission to the clinique, besides the symptoms mentioned, the action of the heart was very violent, its percussion-dullness covered a very wide extent, and a well-marked diastolic bruit was heard along the aorta. Urine scanty, dark yellow, containing much albumen and tube-casts.

About two weeks later, no material change having taken place in the mean while, symptoms of pneumonia in the upper part of the left lung manifested themselves. This condition seemed to be subsiding at the fifth day, and he became able to pronounce a few words distinctly, but on the next day (the twenty-first from the time of his loss of consciousness) he showed signs of exhaustion, sank, and died.

*Autopsy*, thirteen hours after death.—"There were fibrinous clots in the longitudinal and basilar sinuses; the surface of the brain was anæmic, and only here and there a few veins were gorged with blood. There was a considerable

* The time is stated in another place as *ten* hours.

amount of subarachnoid fluid, as well as a good deal in the occipital fossæ. The left middle cerebral artery (*l'artère sylvienne*) was seen to be prominent, solid, as if filled with an artificial injection. Externally it presented the characters of arteritis secondary to its obstruction. The walls, especially the external coat, were thickened, indurated, of a yellowish-gray color, and the cellular tissue surrounding the artery shared in the inflammatory induration. The artery was completely obstructed, both in its horizontal and posterior branches. The coagulation was general in the two branches, but the starting-point in the horizontal branch was an adherent clot, decolorized and firm, about one-fifth of an inch in length and thickness; the more diffused coagulation had joined this clot. In the posterior division, starting from its origin, was found an embolie similar to that in the other branch, but of a yellow color, soft, puriform in the centre; from this there had also proceeded a more extended coagulation. It was everywhere very easy to distinguish the secondary coagulations from the primary embolic clots.

"The middle cerebral artery, when cleared of clots, presented internally a healthy appearance; in particular there was a complete absence of atheromatous degeneration. The external cellular coat, however, had double its natural thickness, owing to a very considerable increase of corpuscles of connective tissue, arranged in groups, and offering the appearance chiefly of nuclei undergoing division and multiplication. There was consequently a secondary plastic inflammation of the walls of the artery. The other intra-cranial arteries were healthy.

"All around the affected artery the cerebral substance was softened, of a pulpy consistence, almost diffluent at places, of a pale-yellow color, but in other places of an almost cheesy appearance; in the peripheral portions the softening was of a pale-red color, with dark points due to little capillary ecchymoses, but there was nowhere any apoplectic extravasation. The softening extended from the surface of the base of the brain through the greater part of the optic thalamus, and through the external and posterior part of the corpus striatum of the left side. The softened part of the brain was found, on microscopic examination, to consist chiefly of granular matter, with numerous spherical granular bodies; very few fibres were recognizable, and these were in a state of fatty metamorphosis; there were none of the elements of blood found. All the rest of the brain was normal; there were no clots in the jugular veins."

In the left lung there was gray hepatization superiorly, and a shading off into red hepatization and the normal state below. The heart was large, and on one of the aortic valves there was an ulceration, "to which was adherent a cylindrical decolorized clot, tolerably firm, presenting a torn appearance at its free extremity, and which had" probably (?) "furnished the clots which obstructed the branches of the middle cerebral artery."

"The kidneys were enlarged; they were five inches long, about two and a half broad, and upwards of an inch thick; the surface was pale, lobulated, and granular; the cortical substance was pale; each kidney contained several old decolorized apoplectic clots, surrounded by a dark, retracted border; in addition, the left kidney contained in one of its calices a little branching yellow calculus."—(*Edinburgh Medical Journal* for August, 1860; from *Gazette Médicale de Paris.*)

II.—*Pneumothorax; Subsequent Death from Dissecting Aneurism of the Aorta.* By W. H. RANKING, M.D.

A tall, thin, delicate-looking young man, aged nineteen, was seized while in church with acute pain in the left side, and dyspnœa. On examination, after his removal to his home, the physical signs elicited were those of pneumothorax; but there was no clue to the cause or rationale of its occurrence. His chest returned to its normal state in about two months, and he resumed his business.

On the fifteenth of May last, (how long after the above attack we are not informed,) after having for several days suffered from occasional pains in the back and loins, and from faintness, he fell down in a fainting state while at his office. He recovered sufficiently to be taken home, undressed himself when there, and got into bed; but soon afterwards his friends, alarmed at hearing him shriek, went into the room and found him dying.

An autopsy was made twenty-four hours after death.—All the viscera were sound. Near the apex of the left lung several air-cells had been ruptured into one, and it seemed probable that the pneumothorax began at this point. The aorta, from about a quarter of an inch above its root, was affected with dissecting aneurism; the inner and middle coats were separated by a layer of blood, as far as the abdominal portion of the vessel. Somewhere,—the precise point could not be detected,—there had been an escape of blood into the surrounding tissues; the areolar tissue lying about the aorta was full of clotted blood as far as the iliac arteries. Blood in a fluid state, to the amount of two quarts, was found in the right pleura; a mass of semicoagulated blood was beneath the left parietal pleura, and blood had been extravasated between the folds of the mesentery, as well as into the pelvic areolar tissue. With the exception of a few specks of atheroma, the lining membrane of the aorta seemed healthy; a small slit, about three-eighths of an inch long, in the upper aspect of the arch, seemed to have been the starting-point of the aneurism.—(*British Medical Journal*, August 25th, 1860.)

III.—*Transposition of the Arteries.* Reported by Dr. J. F. MEIGS to the Pathological Society of Philadelphia.

A male child, born April 30, 1860, seemed healthy and nursed well until the twelfth day, although it did not cry vigorously. It then had what was supposed to be a convulsion, and when seen the next day by Dr. Ellwood Wilson, it was in a state of collapse, with blueness about the mouth and eyes.

Dr. Meigs saw the child on the twenty-seventh day, and noted the following conditions: "The child was small for its age; its lips and mouth were slightly, and but slightly cyanosed; elsewhere the complexion was good. Breathing short, high, and frequent. On uncovering the trunk of the body, it was easy to observe that the act of inspiration lifted up the sternum and other regions of the thorax, while the lateral regions of the chest failed to expand at all scarcely, and the base of the thorax actually became smaller, giving to the waist of the infant the appearance of being drawn in circularly by some internal force, or of being driven in by an exterior pressure. On percussion, the chest was less resonant than natural, on both sides, but especially on the right; the percussion-sound was positively dull on the right side, both before and behind. The vesicular murmur was puerile, except over the right side, where it was feeble.

"Cardiac sounds decidedly louder at right scapula than over left. The cardiac impulse of the left nipple was very indistinct, and the sounds there feeble but natural. The natural tic-tac could be heard. On pressing two fingers lightly to the left of the ensiform cartilage, close to the costal cartilages, a very distinct and quite vigorous impulse could be felt, one much more distinct than at the nipple. At this point a distinct blowing sound attended the systole of the heart. The action of the heart was regular, and the pulse frequent.

"The diagnosis made at the time was: atelectasis of both lungs, of right greater than of left; dilatation with hypertrophy of right ventricle; obstruction of the pulmonary artery; and open foramen ovale."

On the forty-fourth day the child died; its fingers and toes had become livid a day or two previous, and it had been drowsy, but had had no convulsions.

*Autopsy.*—Body small and very thin. Costal cartilages pushed forward, on the right side more than on the left; this gave the thorax a curiously deformed and flattened appearance laterally. Base of chest contracted in all its diameters, as though drawn or driven in.

The sternum being removed, the lungs were seen to be withdrawn, especially below, the heart being almost entirely exposed. The lungs were very small, and mostly paler than natural. The anterior halves of both lower lobes were firm, of a dark, purplish tint, and evidently contained no air; their posterior halves contained some air, but were firmer and less crepitating than normal. The upper lobes were healthy. By means of a blow-pipe the lungs were inflated to a very much larger size; they assumed a light, pinkish hue, and became light and elastic.

The heart was a very large one for so small a child. All its cavities were full, especially the right auricle, of soft, black coagula. The walls of the right ventricle were very thick; thicker than those of the left. The foramen ovale presented at its lower part an opening two or three lines in diameter. The orifices of the vena cava seemed smaller than usual.

The aorta and pulmonary artery were transposed. The aorta arose from the right ventricle in the usual position of the pulmonary artery; the pulmonary artery arose from the left ventricle, and, passing under the arch of the aorta, gave to the latter, just beyond the left subclavian, the ductus arteriosus, which was quite pervious and of considerable size. The valves of the heart were healthy, and not transposed.

Dr. Meigs remarks upon the comparative prominence here of the symptoms of atelectasis of the lungs, the cyanosis, although the heart was strikingly malformed, having been but slight. But for the pervious condition of the foramen ovale and the ductus arteriosus, the two circulations would have been entirely distinct, and extra-uterine life would have been impossible for more than a very few moments. Two views have been held as to the cause of cyanosis, some authors ascribing it to stasis of venous blood, and others to the admixture of venous and arterial blood. But in the case just mentioned there was very complete admixture, while the cyanosis was slight. There was a venous stasis, as shown by the systolic blowing sound, due to tricuspid regurgitation; by the powerful impulse of the right ventricle, as well as by its hypertrophied state; and by the dilatation of the right auricle with the open foramen ovale. Dr. Meigs regards the systemic capillaries as the seat of the obstruction in this case, it being well known that highly-carbonized blood circulates in them with difficulty.—(*American Journal of Medical Sciences*, October, 1860.)

IV.—*Cancerous Degeneration of Lung.* Reported by DR. EUGENE PEUGNET to the New York Pathological Society.

The patient, an Irish waiter, aged twenty-eight, unmarried, was admitted into the Bellevue Hospital, New York, January 18, 1860. For eight years he had had severe pain in the *right* side of the chest, causing dyspnœa. Six months before his admission he had noticed a small lump on the *left* side, about the level of the convexity of the eighth rib; it was the seat of intermittent and lancinating pain, and caused dyspnœa. He was cachectic and emaciated in appearance, but had no cough or expectoration. A large tumor was seated on the left side, extending from the fifth to the eighth rib, and measuring four inches antero-posteriorly; the veins on its surface were enlarged, and the intercostal spaces obliterated. Only the right half of the chest expanded in inspiration; no respiratory murmur in left lung. Cough came on about the twenty-second of February, and continued until his death, on the sixth of April. The left side of his face and his left arm were œdematous for a few days before he died.

"*Autopsy, eight hours after death.*—Rigor mortis well marked. Brain not examined. Right lung normal; slight serous effusion in right pleural cavity; heart dislocated to right; some slight evidences of pericarditis, but otherwise healthy. Left lung was found to be degenerated into a cancerous mass, there being no trace of lung tissue left. The diseased lung communicated with the external tumor by means of the fifth, sixth, and seventh intercostal spaces. The ribs were intact. The diaphragm was pushed downward by the diseased mass; the liver was displaced, and in a cirrhosed condition. The kidneys, spleen, pancreas, mesentery, stomach, and intestines were healthy. The external tumor presented a broken-down appearance. On microscopic examination of the specimen, it was found to contain a few cancer-cells, pus, and broken-down granular matter. The specimen taken from the lung contained a greater abundance of cancer-cells."—(*American Medical Times*, July 28, 1860.)

[Interesting as this case is, as above reported, it would have been much more so had the anatomical relations of the mass to the root of the lung, to the ribs, and to the intercostal vessels been detailed; the microscopic characters might also have been described, if not represented.]

V.—*Intestinal Concretions formed of Oat-hairs.* By A. ERNEST SANSOM, M.B., London.

H. Guy, aged twelve, was admitted into King's College Hospital, May 23d, 1860, having been suffering for four days with symptoms of colic; there was some ground for suspecting intussusception. Two days before his admission into the hospital an enema brought away some dark, feculent matter, followed by clotted blood. At the time of his admission he was evidently suffering from peritonitis, which carried him off on the twenty-seventh.

"*Post-mortem examination*—On incising the walls of the abdomen, a quantity of pus was seen lying between and upon the intestines. The visceral peritoneum was much congested, being, over the lower part of the small intestine, of a deep-purple color. The vermiform appendix was purplish black; it was perforated in four places, and was almost entirely in a gangrenous state; in it was impacted a concretion of about the size and form of a date-stone. The contents of the bowels had not escaped into the abdominal cavity.

"On microscopical and chemico-microscopical examination of the concretion, I found it to consist of phosphate of lime, carbonate of lime, and ammoniaco-magnesian phosphate, surrounding a nidus formed of interlacing vegetable hairs; evidently hairs of the oat."

It being ascertained that this boy had been very fond of a certain kind of very coarse plum-pudding, a specimen of the latter was procured and microscopically examined, when hairs were found in it having exactly the appearance of those observed in the concretion. The explanation of the case was therefore very easy.—(*British Medical Journal*, August 25, 1860.)

VI.—*Diseases of the Uterus.* Descriptions of two Specimens by JOHN CLELAND, M.D., Demonstrator of Anatomy in the University of Edinburgh.

1. *Rupture of the Uterus from Malignant Disease.*—In a female subject about fifty years of age, a wound was observed on the anterior wall of the uterus; it was three inches long, straight and symmetrical, and situated a little above the line between the uterus and bladder. "On more careful examination, it was found that the interior of the uterus was hollowed out into a large cavity by malignant disease. Not a vestige of the cervix was left; but the vagina, which was quite healthy, was continued at once, without any constriction, into this ulcerated cavern. The disease appeared to have quitted, in great measure, the posterior wall of the uterus; for most of that part was comparatively smooth, with a little shredded texture on its surface, but without alteration of the subjacent tissue. On the other hand, the anterior wall and fundus were covered by a growth of ragged villous appendages, which constituted the greater part of their thickness. The greater number of these villi were about a line long, and formed a thick carpet; but a number of others, branching, and about four times as long, were interspersed among them. Professor Simpson recognized this appearance as characteristic of malignant disease. The rupture into the peritoneum extended from side to side of the enlarged uterine cavity, and was about three-quarters of an inch above the termination of the vagina.

"But the disease had also made other perforations. It had distended the uterus so far that the ovaries were brought into contact with the uterine wall; and on the right side was a small perforation from the cavity of the uterus, opening into the peritoneum between the ovary and the Fallopian tube; while on the left side was a perforation as large as a sixpence beneath the ovary. Another small perforation was found, which passed from the back of the uterus into the recto-vesical fossa toward the right side, and was concealed by the way in which the rectum lay in relation to the uterus. More important than any of these, in the deeper part of the lower lip of the great rupture, was observed a lacerated opening into the bladder, so large that a shilling might have been made to pass through it. Notwithstanding all these lesions, there was no appearance of peritonitis."

2. *Disease of the Ovaries, with Peritoneo-rectal Fistula.*—"The subject in which the condition of parts about to be described occurred was the body of a woman between fifty and sixty years old. When the peritoneal cavity was opened, the uterus and ovaries were observed to be altered and enlarged by disease; and the forefinger, on being placed between the uterus and rectum, came in contact with a calcareous body lying toward the left side, quite bare of peritoneum, but concealed from sight by the position of the uterus and appendages. * * * * The uterus was swollen to more than twice its normal dimensions; its walls, on its being opened, presented a thickness varying from a quarter to half an inch; and the cavity was gorged with a thick, white substance, of almost cheesy consistence, which in its physical characteristics resembled tubercular deposit. No trace of the mucous membrane of the uterus remained; the boundaries of the uterine cavity were not defined; but the substance which filled the centre was infiltrated among the deeper fibres of the walls. The os uteri and vagina were healthy; and from the former depended a small quantity of tough, clear mucus.

"The Fallopian tubes were, at their origins, hypertrophied to a diameter of four lines, and looked like the cornua uteri of some lower animal. Their thickness was entirely due to development of fibres. On incisions being made into them, they were found to be quite solid, and no traces were found where the canals had been.

"On the right side of the pelvis, in contact with the uterus, was a large, hard tumor, about the size and of the form of a turkey's egg, with the broad end foremost; only it was somewhat curved upon itself, as if to adapt it to the shape of the pelvis, as its long axis lay horizontally. This tumor presented, over more than half its surface, a free aspect invested with peritoneum; and cul-de-sacs of the serous membrane, which could be followed out by cutting short folds that bound the tumor to its position, showed that it had originally presented a much larger free surface. On cutting into it, it was found to be enveloped by fibrous tissue; and the deeper part of this investment formed a separate layer, firm and tough like fibro-cartilage, but entirely fibrous in structure. Inside this tunic, intimately connected with it, was an imperfect calcareous shell, of almost bony consistency, extended in an irregular fashion over the contents of the tumor, but leaving large spaces uncovered. The fibrous tunic was thinned away in the places where the calcareous shell was thickest, as though the latter had been formed at the expense of the former. The interior was filled with a mass of substance having very much the appearance and somewhat the smell of half-dry putty. It crumbled down in irregular masses, and in powder, when disturbed. Nothing could be made of it by microscopic examination. A small portion shaken with ether was not visibly lessened in bulk. On the upper anterior part of the tumor, the remains of the ovary were discovered on dissection. It was joined to the uterus by a much hypertrophied ligament about half an inch long, whose fibres in great part spread out and entered into the formation of the wall of the tumor. The Fallopian tube of this side was hypertrophied, in the manner already described, for not more than half an inch of its extent; then became abruptly converted into a fibrous band about one and a half inch long, which was folded on itself, and hid from view between the tumor and uterus, terminating on the part of the tumor nearest the uterus.

"On the left side the Fallopian tube continued hypertrophied, and curved outward and backward for nearly two inches, forming the anterior margin, and incorporated in the formation of a cup, in which lay loose the body which had been felt in the peritoneal cavity before the removal of the parts from the pelvis. This body was about the size of a walnut. It consisted of a calcareous shell, corresponding to that of the tumor of the right side, and nearly perfect on the aspect which had lain uncovered, but very incomplete on the aspect which had occupied the base of the cup-like cavity. The contents were soft and slimy, with a disposition in part to cohere. The shell was structureless, as seen on examination of a portion under the microscope. It is impossible to say definitely how the Fallopian tube terminated on this side. The cup which contained the tumor was bounded behind by the rectum, and presented two different appearances in its floor. The outer half had a smooth surface broken up into convoluted elevations, like those of the brain in miniature. The inner and deepest part, lying between the rectum and posterior wall of the uterus, presented no such convolutions, and had a rough, shredded, and ulcerated appearance. On opening the rectum, it was found that a fistulous communication, which admitted a porcupine's quill, passed from the deepest ulcerated portion of the cup downward into the rectum, between two and three inches above the anus. This passage was at least a quarter of an inch long, bounded by strong fibrous walls; and its opening into the rectum was drawn up in such a way as to be overhung by a fold immediately above it—a circumstance calculated to protect it from the ingress of fecal matters."

